# Frequency and Env determinants of HIV-1 subtype C strains from antiretroviral therapy-naive subjects that display incomplete inhibition by maraviroc

**DOI:** 10.1186/s12977-016-0309-2

**Published:** 2016-11-03

**Authors:** Katharina Borm, Martin R. Jakobsen, Kieran Cashin, Jacqueline K. Flynn, Paula Ellenberg, Lars Ostergaard, Benhur Lee, Melissa J. Churchill, Michael Roche, Paul R. Gorry

**Affiliations:** 1 Center for Biomedical Research, Burnet Institute, Melbourne, VIC Australia; 2 Department of Microbiology, La Trobe University, Melbourne, VIC Australia; 3 Department of Biomedicine, Aarhus University, Aarhus, Denmark; 4 School of Health and Biomedical Sciences, College of Science, Engineering and Health, RMIT University, Melbourne, VIC 3001 Australia; 5 Department of Infectious Diseases, Monash University, Melbourne, VIC Australia; 6 Icahn School of Medicine at Mount Sinai, New York, NY USA; 7 Department of Medicine, Monash University, Melbourne, VIC Australia; 8 Department of Microbiology, Monash University, Melbourne, VIC Australia; 9 The Peter Doherty Institute for Infection and Immunity, University of Melbourne and Royal Melbourne Hospital, Melbourne, VIC 3000 Australia

**Keywords:** HIV-1, Subtype C, Env, gp120, CCR5, Maraviroc

## Abstract

**Background:**

Entry of human immunodeficiency virus type 1 (HIV-1) into cells involves the interaction of the viral gp120 envelope glycoproteins (Env) with cellular CD4 and a secondary coreceptor, which is typically one of the chemokine receptors CCR5 or CXCR4. CCR5-using (R5) HIV-1 strains that display reduced sensitivity to CCR5 antagonists can use antagonist-bound CCR5 for entry. In this study, we investigated whether naturally occurring gp120 alterations in HIV-1 subtype C (C-HIV) variants exist in antiretroviral therapy (ART)-naïve subjects that may influence their sensitivity to the CCR5 antagonist maraviroc (MVC).

**Results:**

Using a longitudinal panel of 244 R5 Envs cloned from 20 ART-naïve subjects with progressive C-HIV infection, we show that 40% of subjects (n = 8) harbored viruses that displayed incomplete inhibition by MVC, as shown by plateau’s of reduced maximal percent inhibitions (MPIs). Specifically, when pseudotyped onto luciferase reporter viruses, 16 Envs exhibited MPIs below 98% in NP2–CCR5 cells (range 79.7–97.3%), which were lower still in 293-Affinofile cells that were engineered to express high levels of CCR5 (range 15.8–72.5%). We further show that Envs exhibiting reduced MPIs to MVC utilized MVC-bound CCR5 less efficiently than MVC-free CCR5, which is consistent with the mechanism of resistance to CCR5 antagonists that can occur in patients failing therapy. Mutagenesis studies identified strain-specific mutations in the gp120 V3 loop that contributed to reduced MPIs to MVC.

**Conclusions:**

The results of our study suggest that some ART-naïve subjects with C-HIV infection harbor HIV-1 with reduced MPIs to MVC, and demonstrate that the gp120 V3 loop region contributes to this phenotype.

**Electronic supplementary material:**

The online version of this article (doi:10.1186/s12977-016-0309-2) contains supplementary material, which is available to authorized users.

## Background

Entry of human immunodeficiency virus type 1 (HIV-1) into cells is mediated by the envelope glycoprotein complex (Env). Env exists as a trimer of heterodimers of gp120 surface and gp41 transmembrane glycoproteins that decorate the exterior of the virus particle (reviewed in [1]). Entry is initiated by the interaction between gp120 and cell surface CD4 [2–4], followed by a secondary interaction with a chemokine coreceptor, either CCR5 or CXCR4 [5–9]. HIV-1 Envs are classified as CCR5-using (R5) or CXCR4-using (X4) depending on the choice of coreceptor used for entry. HIV-1 Envs that can interact with either CCR5 or CXCR4 to enter cells are referred to as dual-tropic (R5X4).

The interaction between gp120 and CD4 leads to conformational changes in gp120 affecting the first, second and third variable loops (V1, V2, V3) and formation of the gp120 bridging sheet, which together comprise the coreceptor binding site (CoRbs) [10–12]. CCR5 is a G-protein coupled receptor that contains an N-terminal domain and 7 transmembrane helices, the latter of which forms a hydrophobic cavity at the cell membrane [13, 14]. The stem of the gp120 V3 loop and the bridging sheet of CD4-bound gp120 interact with the N-terminal domain of CCR5, and the tip of the V3 loop interacts with the second extracellular loop (ECL2) of the coreceptor [15–17]. After coreceptor binding, further conformational rearrangements in gp41 occur that lead to virus-cell fusion and release of the viral core into the cell.

Maraviroc (MVC) is a CCR5 antagonist that is approved for use as an antiretroviral drug in treatment-experienced and -naïve HIV-1 infected subjects shown to harbour only R5 viruses [18–20]. Furthermore, since R5 HIV-1 strains are preferentially transmitted from person to person, MVC has potential for use in pre-exposure prophylaxis (PrEP) and microbicide formulations to prevent new infections [21–23]. Other experimental CCR5 antagonists that are not used clinically include vicriviroc (VVC) and aplaviroc (APL). MVC and other CCR5 antagonists inhibit HIV-1 entry by binding within the hydrophobic pocket of CCR5 [24–26], thereby altering the conformation of the CCR5 extracellular loops [27] such that they are no longer recognized by gp120. Consequently, MVC and other CCR5 antagonists are allosteric inhibitors of HIV-1 entry rather that competitive inhibitors.

Some HIV-1 infected subjects experience resistance to MVC after treatment with MVC-containing ART regimens [18, 19]. There are two principal mechanisms that contribute to the emergence of MVC-resistant HIV-1 strains in these subjects; (1) a selection for minor X4 or R5X4 HIV-1 strains that are not inhibited by CCR5 antagonists [19] or (2) acquisition of the ability of R5 viruses to interact with the MVC-bound conformation of CCR5 [28–30]. In the latter scenario that typifies “genuine” MVC resistance, the use of MVC-bound CCR5 by resistant viruses is characterized not by increases in the 50% inhibitory concentrations (IC_50_) of MVC, but rather by reductions in the maximal percent inhibition (MPI) in viral infection assays, with plateaus of incomplete inhibition being evident despite saturating drug concentrations [28–30]. Considering the extensive variability of Env sequence between HIV-1 strains, and the inherently flexible nature of coreceptor engagement by Env, we hypothesized that the existence of clinical HIV-1 strains isolated from ART-naïve subjects that exhibited reduced MPIs to MVC was likely [31, 32]. In fact, recent studies have shown that Envs cloned from viruses isolated from treatment-naïve individuals chronically infected with HIV-1 subtype C (C-HIV), may be more efficient at interacting with MVC bound CCR5 than Envs from viruses isolated from subjects with acute infection [33, 34]. However, usage of MVC-bound CCR5 by viruses characterized in these studies was generally very inefficient compared to that which occurs in genuinely resistant HIV-1 strains isolated from patients failing MVC therapy. Whether such baseline usage of MVC-bound CCR5 develops further at late stages of infection and occurs at potentially clinically relevant efficiencies is yet to be determined.

Globally C-HIV is the most rapidly spreading HIV-1 subtype, yet paradoxically, it is less virulent than other subtypes ex vivo [35, 36], suggesting unique molecular mechanisms that simultaneously impair fitness and facilitate favourable transmission events. A number of these unique features appear to involve the Env glycoproteins and entry mechanisms. For example, in contrast to HIV-1 subtype B infection where, if untreated, progression to advanced stages of infection is frequently accompanied by a switch in coreceptor usage from R5 to R5X4 or X4 variants (reviewed in [37]), in C-HIV infection the detection of R5X4 and X4 variants is relatively rare, even at late stages of infection ([38] and references within). Furthermore, R5 C-HIV strains have been shown to exhibit alterations in their efficiency of CCR5 and CD4 usage [39], and usage of particular alternative coreceptors in vitro [40–42]. Together, these features of may influence how C-HIV strains respond to inhibition by CCR5 antagonists such as MVC.

In this study we investigated the frequency, efficiency and underlying Env determinants of HIV-1 strains with reduced MPIs to MVC within an ART-naïve, longitudinal cohort of 20 subjects who progressed from chronic to late stages of C-HIV infection over a 3 year period [38]. By functionally characterizing 244 independent Envs derived from plasma of these subjects, we demonstrate that 8 subjects (40%) harbored HIV-1 strains that could interact with MVC-bound CCR5 resulting in reduced MPIs to MVC. We further show that these variants have strain-specific mutations in the gp120 V3 loop region that contribute to this phenotype. However, strains with reduced MPIs to MVC did not emerge more frequently at advanced infection compared to chronic infection, suggesting that they are not selected for during disease progression. Together, our findings suggest that some ART-naïve subjects infected with C-HIV harbor viruses that display altered sensitivity to MVC, which involves strain-specific amino acid alterations the gp120 V3 loop region of Env.

## Methods

### Ethics statement

Written informed consent was provided by the subjects for the use of stored plasma samples (from which the Env clones were derived), as stated previously [38]. Ethics approval for the use of these samples was granted by the Medical Research Council of Zimbabwe (MRCZ/A/918) and by the Central Medical Scientific Ethics Committee of Denmark (624-01-0031).

### Cells

293T cells, and NP2-CD4/CCR5 cells [43] were maintained in Dulbecco’s Modified Eagles Medium (DMEM) supplemented with 10% (vol/vol) fetal calf serum (FCS) and 100 μg/ml of penicillin and streptomycin. CD4 selection in NP2 cells was maintained by 500 μg/ml of G418 and CCR5 expression was maintained by 1 µg/ml of puromycin. 293-Affinofile cells [44] were maintained in DMEM supplemented with 10% (vol/vol) FCS, 100 μg/ml of penicillin and streptomycin, 50 μg/ml of blasticidin and 200 μg/ml of G418.

### Env clones

The sequences of the C-HIV Env clones used here have been reported previously [38], and their GenBank accession numbers are shown in Additional file 1: Table S1.

### Production and titration of Env-pseudotyped luciferase reporter viruses

Env-pseudotyped luciferase reporter viruses were produced by transfection of 293T cells with pCMVΔP1ΔenvpA, pHIV-1Luc, and pSVIII-Env plasmids using lipofectamine 2000 (Invitrogen) at a ratio of 1:3:1, as described previously [45, 46]. Supernatants were harvested 48 h later, filtered through 0.45 μM-pore size filters, and stored at −80 °C. The infectivity of virus stocks was determined by titration in NP2-CD4/CCR5 cells. For subsequent infection experiments of all cell lines, viral input was normalized to 2 × 10^5^ relative light unit (RLU) counts.

### HIV-1 inhibition assays

The sensitivity of Env-pseudotyped luciferase reporter viruses to inhibition by MVC was determined as described previously [29, 47, 48]. Briefly, to obtain 293-Affinofile cells with a medium/high level of CD4 and high level of CCR5 (termed CD4^med^/CCR5^hi^ cells), 293-Affinofile cells were induced for 20 h prior to seeding with 2.5 ng/ml of minocycline (for CD4 expression) and 2.0 μM of Ponasterone A (for CCR5 expression). In our hands this produces a cell type that expresses approximately 180,000 molecules per cell of CD4 and approximately 95,000 molecules per cell of CCR5, as determined by quantitative flow cytometry (qFACS) as described previously [49]. NP2-CD4/CCR5 cells and 293-Affinofile CD4^med^/CCR5^hi^ cells (1 × 10^4^ in 100 μl) were seeded in flat-bottom 96-well plates. 24 h later the media was removed from cells and replaced with 100 µl of fresh media with fivefold dilutions of MVC (at a × 2 concentration) for 30 min at 37 °C. The concentration range for MVC was 5 μM to 0.064 nM. NP2-CD4/CCR5 cells and 293-Affinofile CD4^med^/CCR5^hi^ cells were inoculated with Env-pseudotyped luciferase reporter viruses in 100 μl for 12 h at 37 °C. Following this, the inoculum was removed and replaced with fresh media. The inhibitor concentrations were maintained throughout the subsequent culture period. Infected cells were incubated at 37 °C for a total of 72 h. The level of HIV-1 entry was measured by luciferase activity in cell lysates (Promega) according to the manufacturers protocol. Luminescence was measured using a FLUOStar microplate reader (BMG). Background activity was assessed by mock-infected cells and was subtracted from all wells. The amount of luciferase activity in cells treated with MVC was expressed as a percentage of that in untreated cells. The percentage of inhibition was calculated by subtracting this number from 100. The data were fitted with a nonlinear function, and alterations in inhibitor sensitivity were assessed by reductions in the MPI as described previously [47].

### Measurement of CD4/CCR5 usage efficiency

293-Affinofile cells were infected with Env-pseudotyped luciferase reporter viruses as described previously [29, 49]. Briefly, 48 populations of cells expressing different combinations of CD4 and CCR5 levels were generated by inducing cells with twofold serial dilutions of minocycline (0.156–5.0 ng/ml) and ponasterone A (0.0156–2.0 μM). CD4 and CCR5 concentrations were determined by quantitative flow cytometry (qFACS) as described previously [44, 50]. CD4 expression ranged from 1600 to 190,000 molecules per cell; CCR5 expression ranged from 1500 to 95,000 molecules per cell. The induced cell populations were then either left untreated or treated with 10 μM MVC for 30 min at 37 °C, after which they were inoculated with 2 × 10^5^ RLU of Env-pseudotyped luciferase reporter viruses and were analyzed for levels of HIV-1 entry 72 h later as described above. In experiments using MVC-treated cells, the MVC concentration was maintained throughout the culture period. The relative level of virus entry achieved by each Env was expressed as a percentage of that achieved in 293-Affinofile cells expressing the highest concentrations of CD4 and CCR5. For infections performed in the presence of MVC, virus entry was expressed as a percentage of that achieved in untreated wells.

### Env mutagenesis

Env mutants were generated with a Quick Change II site-directed mutagenesis kits (Agilent Technologies) according to the manufacturers’ protocol, and were verified by full-length sequencing.

## Results and discussion

### Detection of incomplete inhibition by MVC in HIV-1 strains from a treatment-naïve, longitudinal cohort of subjects with progressive HIV-1 subtype C infection

Recent cross sectional studies demonstrated levels of incomplete inhibition of C-HIV strains by MVC that differed between transmitted and chronic Envs [33, 34]. However, the levels of incomplete inhibition were generally relatively low, with the majority of Envs tested exhibiting residual entry levels of just 0.01–1.0% in NP2-CD4/CCR5 cells in the presence of a saturating concentration of MVC [34]. Since the threshold for the laboratory diagnosis of clinical MVC-resistance in similar cell lines (U87-CD4/CCR5) is typically at 5% residual entry or above in the presence of MVC [51], whether levels of residual entry below 5% are clinically relevant remains to be determined.

**Fig. 1:**
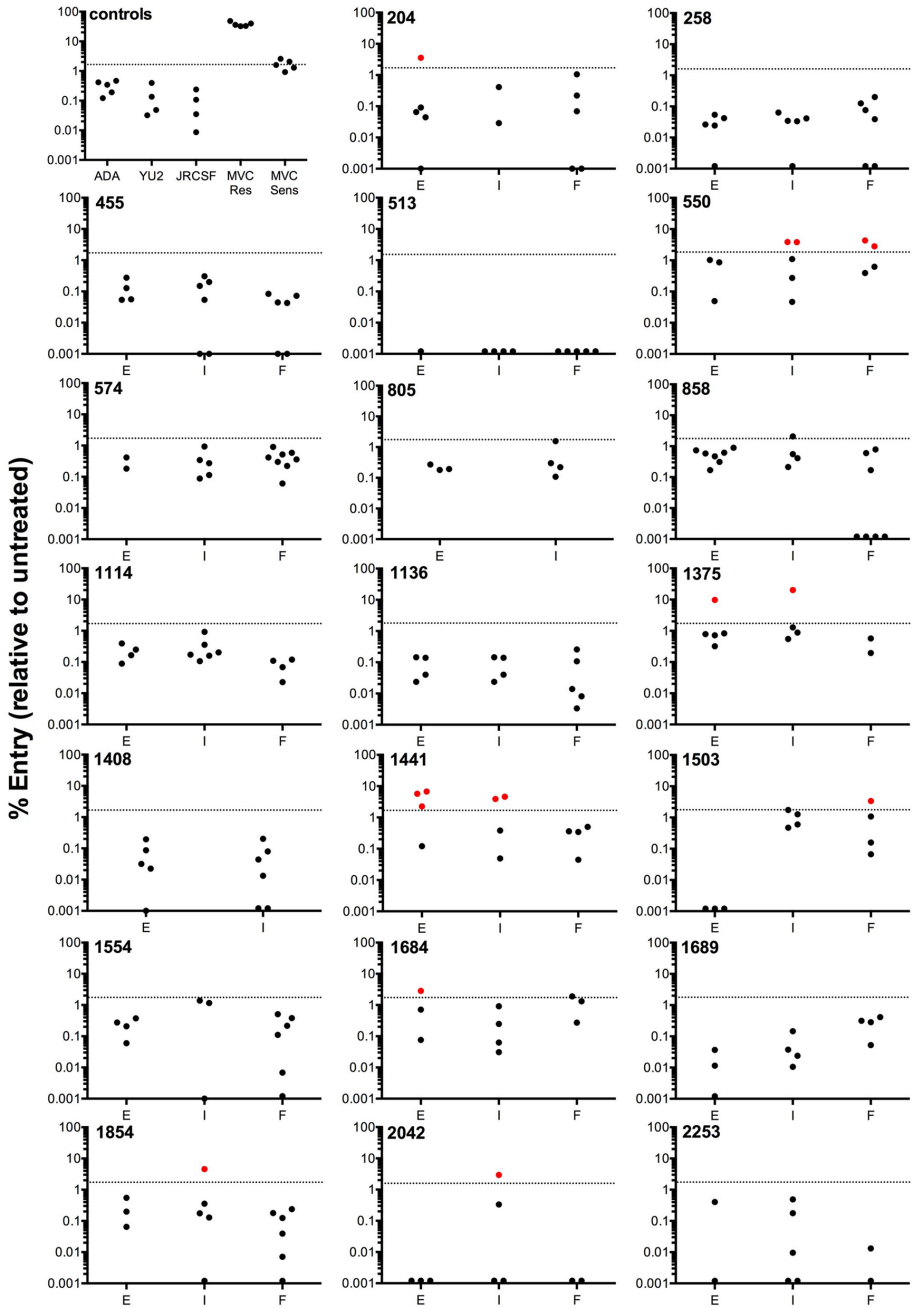
Residual viral entry in the presence of saturating MVC in NP2-CD4/CCR5 cells infected with HIV-1 subtype C Envs from a longitudinal progressor study. Luciferase reporter viruses pseudotyped with HIV-1 subtype C Envs were used to infect NP2-CD4/CCR5 cells in the presence of saturating amounts of MVC. Residual entry was expressed as the percentage of entry achieved compared to no drug controls. The data points are means of triplicate wells. *Dotted line*, arbitrary cutoff value set by MVC-Sens residual entry; *red dots*, clones above cutoff and thus considered resistant; *black dots*, clones below cutoff and thus considered sensitive. *E* enrolment timepoint, *I* intermediate timepoint, *F* final timepoint

To better understand the frequency and magnitude of residual entry by C-HIV strains in the presence of MVC that may arise in untreated subjects during progressive infection, we first screened the infectivity of 244 R5 C-HIV Envs that were isolated from longitudinal plasma samples of 20 ART-naïve subjects from rural Zimbabwe. These subjects progressed from chronic to advanced stages of infection over a 3 year period [38]. Figure 1 shows the percentage of residual entry of Env-pseudotyped luciferase reporter viruses when inoculated onto NP2-CD4/CCR5 cells in the presence of saturating MVC (5 µM). For these and subsequent results, “E” refers to Envs cloned from plasma taken at study enrolment, “I” refers to Envs cloned at an intermediate sampling timepoint 1 year after study enrolment, and “F” refers to Envs cloned at the final timepoint 3 years after study enrolment [38]. Controls included (1) luciferase viruses pseudotyped with the R5 Envs ADA, YU2 or JRCSF, which as expected, exhibited levels of residual entry <0.04% indicative of MVC sensitivity; (2) virus pseudotyped with a MVC-resistant Env that was generated in vitro (MVC-Res) [48] which, consistent with the results of previous studies [47] exhibited levels of residual entry between 30 and 40%; and (3) virus pseudotyped with the parental Env to which MVC-Res was derived (MVC-Sens), which we have previously shown has a degree of inherent baseline ability to interact with MVC-bound CCR5 [47], and consistent with those studies exhibited levels of residual entry ~2%. From the results of our control infections, and in consideration of thresholds applied for MVC-Sens Env, we applied a cutoff of >2% residual entry in these analyses for the identification of C-HIV Env clones that may exhibit residual entry levels that could potentially be clinically relevant.

Analysis of viruses pseudotyped with the C-HIV Envs showed that 8/20 subjects (40%) had Env variants that displayed residual entry levels >2% in the presence of saturating MVC (Fig. 1). Residual entry levels ranged from 2.7 to 20.3% (Table 1). Overall, there was no temporal pattern for emergence of Envs displaying residual entry in the presence of MVC, with these Envs detectable within early, intermediate and final plasma samples. However, the number of Env clones tested at each of the timepoints was relatively small, so further studies of greater numbers of Env clones may be required to observe a relationship between Envs displaying a residual entry phenotype and disease stage.

**Table 1:** Residual entry of selected HIV-1 subtype C Envs in the presence of MVC in NP2-CD4/CCR5 and 293-Affinofile CD4^med^/CCR5^hi^ cells

Patient ID	Env clone	Residual entry in NP2 cells (%)^a^	Residual entry in Affinofile cells (%)^a^
	ADA	0.25 ± 0.04	n.a.
	YU2	0.04 ± 0.02	0.26 ± 0.02
	JRCSF	0.09 ± 0.02	0.18 ± 0.02
	MVCres	37.8 ± 1.01	68.45 ± 14.35
	MVCsens	1.82 ± 0.2	47.28 ± 2.23
204	E-7	3.6 ± 0.09	36.24 ± 5.01
550	I-2	3.80 ± 0.71	52.6 ± 13.87
	I-7	3.84 ± 1.00	36.2 ± 1.81
	F-12	4.32 ± 1.32	60.32 ± 7.20
	F-20	2.80 ± 1.11	38.83 ± 4.09
1375	E-2	9.73 ± 2.50	84.20 ± 8.99
	I-8	20.34 ± 5.23	69.03 ± 9.10
1441	E-1	5.70 ± 0.47	44.77 ± 2.55
	E-2	2.7 ± 1.00	53.69 ± 9.14
	E-6	6.72 ± 0.66	67.26 ± 9.58
	I-2	3.90 ± 0.49	68.34 ± 11.52
	I-9	4.57 ± 0.42	79.78 ± 4.18
1503	F-7	3.37 ± 0.71	n.a.
1684	E-7	2.84 ± 1.74	27.46 ± 3.51
1854	I-7	4.56 ± 0.73	n.a.
2042	I-38	2.95 ± 0.86	38.77 ± 0.53

*n.a.* not done

^a^Mean % residual entry ± standard deviation from two independent experiments

Together, our results show that a proportion of C-HIV Envs from our cohort is capable of mediating entry into NP2-CD4/CCR5 cells in the presence of MVC. Our results confirm the observations of others who have found a portion of acute disease-derived and chronic disease-derived Envs with similar phenotypes [33, 34]. Indeed both Parker et al. and Ping et al. found the residual entry in the presence of saturating MVC to be more frequent in chronic disease-derived Envs. We have extended these observations by showing that this phenomenon does not appear to increase during the course of active disease as we found no difference in the proportion of the residual entry phenotype between the enrolment, intermediate and final timepoints. Interestingly, we have found that, at a population level, the ability of our panel of C-HIV Envs to utilise the alternative coreceptors CCR3 and FPRL1 increased as disease advanced suggestive of alterations in the flexibility of Env to engage coreceptor [40]; the individual alternative coreceptor usage characteristics of the Envs that display entry in the presence of MVC, in comparison to a representative Env from each subject that is completely inhibited by MVC, is shown in Additional file 2: Table S2. Increased flexibility in coreceptor engagement has been observed previously in studies of disease progression in subtype B infection [52, 53], and this has been associated with reduced sensitivity to RANTES and TAK-779 as determined by increases in IC_50_ values [53, 54]. Recent evidence suggests that this is also the case for MVC [55]. Interestingly, MVC resistance is commonly associated with increased Env dependence on interactions with the CCR5 N-terminus, likely signalling a movement toward regions of CCR5 not modified by MVC binding [29, 30, 48]. Conversely, reduced sensitivity to MVC, as determined by increases in IC_50_/IC_90_, is associated with reduced dependence on the CCR5 N-terminus [31, 55]. Taken together, our results suggest that increased flexibility of CCR5 usage may not necessarily lead to strains with reduced MPIs to MVC.

Whilst our data demonstrates the presence of C-HIV Envs with baseline reductions in MPI to MVC, there is evidence that this phenotype is not unique to C-HIV. Parker et al. observed similar levels of reduced baseline MPI in chronic-derived Envs from both B- and C-HIV [56]. In addition, the MVC-Sens control Env for our assays that displays reduced baseline MPI, is derived from the CC1/85 isolate, itself a B-HIV strain [28]. Thus, the phenomenon of baseline MPI reductions to MVC is unlikely to be limited to C-HIV and may be a more general feature of chronic derived Envs.

### Levels of incomplete HIV-1 inhibition by MVC are more pronounced in 293-Affinofile CD4^med^/CCR5^hi^ cells

We have previously demonstrated that obscure or borderline MVC resistance profiles seen in U87-CD4/CCR5 and NP2-CD4/CCR5 cells are amplified in 293-Affinofile cells [47]. This may be related to a higher level of CCR5 expression on 293-Affinofile cells engineered to express high levels of CCR5, or expression of conformations of CCR5 not bound by MVC [33, 47]. We therefore conducted HIV-1 entry experiments in 293-Affinofile cells that were induced to express moderate CD4 levels and high CCR5 levels (293-Affinofile CD4^med^/CCR5^hi^), in the presence of saturating MVC (10 μM). Table 1 compares the residual entry in the presence of MVC between experiments conducted in NP2-CD4/CCR5 cells and 293-Affinofile CD4^med^/CCR5^hi^ cells. The residual entry levels in 293-Affinofile CD4^med^/CCR5^hi^ cells by the Envs tested ranged from 27.5 to 84.2%, which are values approximately 7- to 20-fold greater than those derived from the NP2-CD4/CCR5 cell experiments. Importantly, control experiments with viruses pseudotyped with YU2 and JRCSF Envs demonstrated residual entry levels <0.5% in 293-Affinofile CD4^med^/CCR5^hi^ cells, confirming that 293-Affinofile cells have greater sensitivity for detection of HIV-1 strains that are incompletely inhibited by MVC without compromising specificity.

**Fig. 2:**
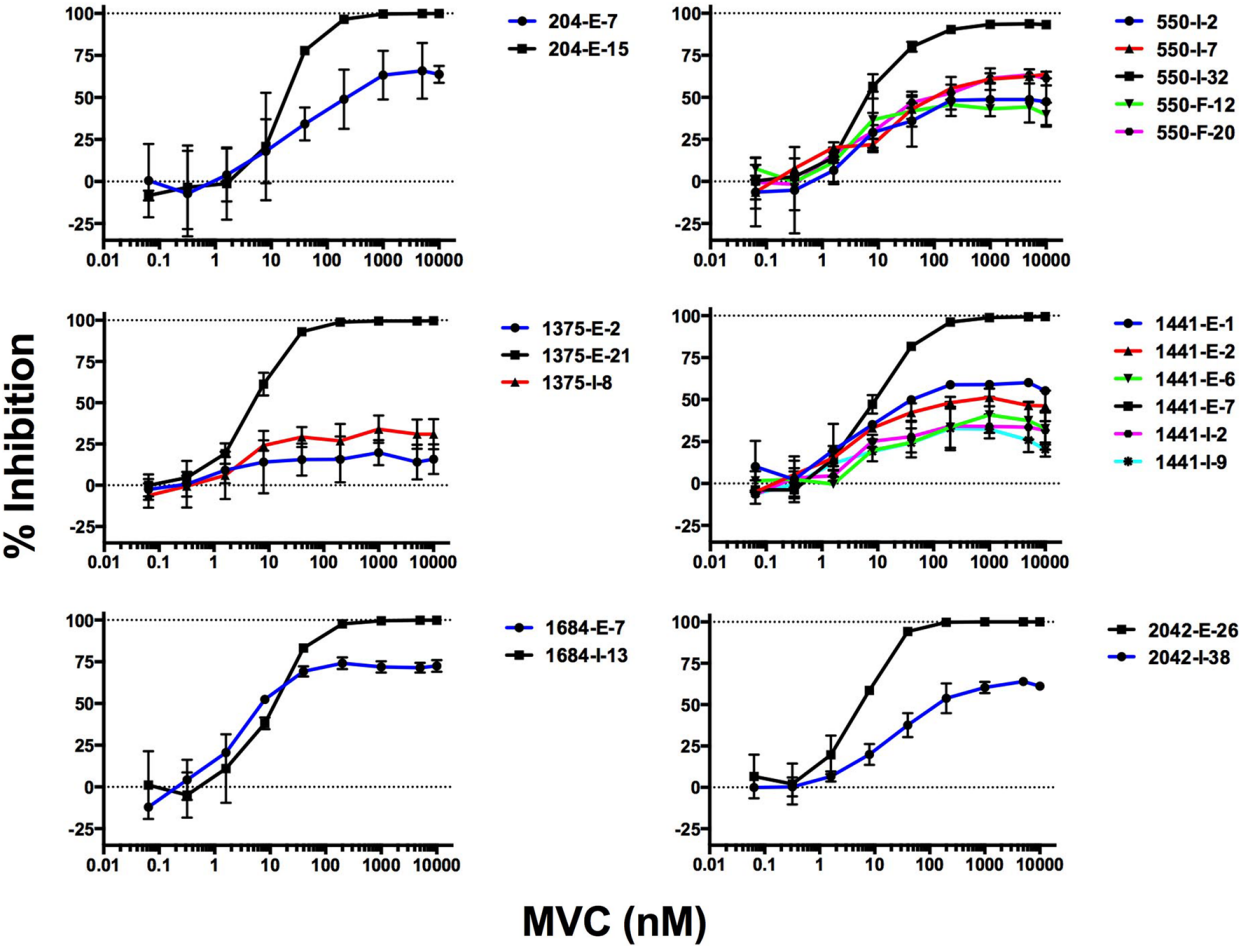
MVC sensitivity curves in 293-Affinofile CD4^med^/CCR5^hi^ cells. Luciferase reporter viruses were used to infect 293-Affinofile CD4^med^/CCR5^hi^ cells in the presence of increasing concentrations of MVC. Data points represent the mean and standard error of infections performed in triplicate from one representative experiment. Viral inhibition curves were constructed as described in the “Methods”

Whilst the results of the preceding experiments demonstrate the frequency and magnitude of C-HIV Envs displaying a residual entry despite a saturating concentration of MVC, suggesting usage of MVC-bound CCR5 for entry, *bone fide* MVC resistance is characterized by plateaus of incomplete inhibition in response to escalating drug concentrations [28]. We therefore next performed confirmatory titration experiments on 14 of the incompletely inhibited C-HIV Envs from 6 subjects, in 293-Affinofile CD4^med^/CCR5^hi^ cells, with comparison to a representative Env cloned from the same plasma sample that did not display residual entry in the presence of MVC (Fig. 2). As expected, most of the Envs that did not display residual entry in the presence of MVC achieved MPIs of ~100%; the exception to this was Env 550-I-2, which although completely inhibited by MVC in NP2-CD4/CCR5 cells, displayed an MPI of 92.7% in 293-Affinofile CD4^med^/CCR5^hi^ cells (Fig. 2; Table 2). These results suggest that Env 550-I-2 may also possess a low level of altered MVC sensitivity that was not detected in the NP2-CD4/CCR5 cell screen. All the Envs that displayed residual entry despite MVC in both NP2-CD4/CCR5 cells and 293-Affinofile CD4^med^/CCR5^hi^ cells exhibited plateaus of incomplete inhibition in response to escalating MVC concentrations (Fig. 2), with MPIs ranging from 27.5 to 72.6%. These MPIs are consistent with the definition of “moderate” to “low level” MVC resistance, respectively, that has been determined for viruses isolated from patients failing MVC-containing ART regimens [29].

**Table 2:** Maximal percent inhibition (MPI) of selected HIV-1 subtype C Envs by maraviroc in 293-Affinofile CD4^med^/CCR5^hi^ cells

Patient ID	Env clone	MPI (%)^a^	SD (%)^b^
204	E-15	100.13	1.66
	E-7	68.65	16.62
550	I-32	92.7	0.62
	I-2	49.03	13.75
	I-7	65.38	3.33
	F-12	43.39	1.42
	F-20	62.65	2.09
1375	E-21	100.01	0.69
	E-2	16.54	10.53
	I-8	30.87	9.17
1441	E-7	99.25	0.56
	E-1	60.47	3.94
	E-2	48.02	5.56
	E-6	36.85	12.35
	I-2	32.92	12.34
	I-9	27.51	0.05
1684	I-13	100.27	1.46
	E-7	72.63	2.91
2042	E-26	100.99	1.57
	I-38	63.55	1.30

*MPI* maximal percent inhibition, *SD* standard deviation

^a^Mean and ^b^ standard deviation of three independent experiments

Our results with 293-Affinofile CD4^med^/CCR5^hi^ cells demonstrate a striking phenotypic similarity between the Env clones with reduced MPIs to MVC that we have detected in treatment naïve individuals and Env clones isolated from individuals who have failed MVC therapy [29, 30]. Based on the plateaus in MPI in 293-Affinofile CD4^med^/CCR5^hi^cells, the Env clones with reduced MPIs to MVC characterized here are likely interacting with MVC-bound forms of CCR5 and with an efficiency similar to strains with genuine resistance to MVC [29]. Our findings further highlight the efficiency of 293-Affinofile CD4^med^/CCR5^hi^ cells in determining the magnitude of reduced MPIs by strains with altered MVC sensitivity. This may be due to the high levels of CCR5 expression on these cells when maximally induced with Ponasterone, higher than seen on other cell types [34, 47]. At high concentrations of MVC, this would lead to a greater density of MVC bound CCR5 leading to a more efficient interaction between CCR5 and MVC resistant gp120. Conversely, 293-Affinofle cells may express a minority population of CCR5 that cannot be bound by MVC but can be bound by gp120 [33]. This minority species may only become relevant when CCR5 expression is induced to high levels on these cells. The concept of distinct forms of CCR5 expressed on different cell types has been proposed as a reason as to why CCR5 antagonist resistance manifests itself differently between primary CD4+ T cells and engineered cell lines such as TZM-bl [57]. Our results suggest that 293-Affinofile CD4^med^/CCR5^hi^ cells may provide greater sensitivity for the detection of HIV-1 strains that are incompletely inhibited by MVC.

### The magnitude of reduced MPIs to MVC by C-HIV Envs is related to the efficiency of usage of MVC bound CCR5

**Fig. 3:**
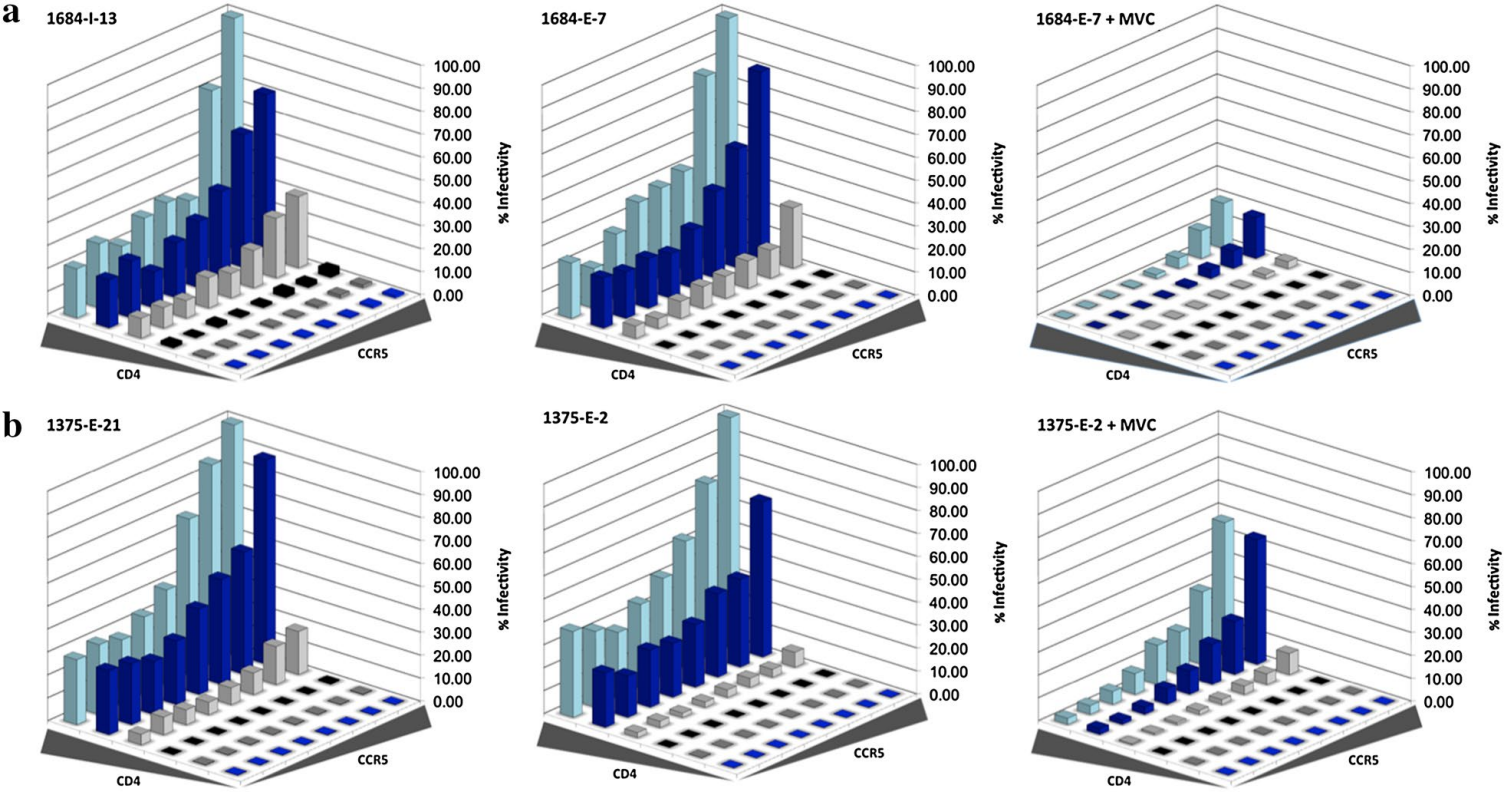
The level of incomplete HIV-1 inhibition by MVC in subtype C Envs is dependent on the efficiency of interaction with MVC-bound CCR5. Luciferase reporter viruses from subjects 1684 (**a**) and 1375 (**b**) were used to infect 48 differentially-induced 293-Affinofile cell populations in the presence and absence of 10 μM MVC as described in the “Methods”. The percent infectivity was normalized that achieved in 293-Affinofile cells induced to express the maximal amount of CD4 and CCR5. The data shown are means of duplicate infections and are representative of three independent experiments

Envs displaying moderate to low level MVC resistance have been shown to interact with the MVC-bound CCR5 complex relatively inefficiently compared to their interactions with drug-free CCR5 [47]. We therefore next used affinity profiling to quantify the ability of two C-HIV Envs, representing those with the highest (1684-E-7; MPI 72.6%) and lowest MPIs (1375-E-2; MPI 16.5%) in response to escalating concentrations of MVC (Table 2), to interact with MVC-bound CCR5. To do this we used the 293-Affinofile affinity profiling system where CD4 and CCR5 levels are controlled by separate inducible promoters, permitting independent variation of CD4 and CCR5 expression over a physiological concentration range [50]. When 48 differentially induced cell populations are subjected to entry assays with Env pseudotyped luciferase viruses, relative efficiencies of CD4- and CCR5-usage can be inferred. Results of the affinity profiling experiments are shown in Fig. 3, and illustrate that in the absence of MVC, the Envs that are completely- or incompletely inhibited by MVC from both subjects have similar infectivity profiles suggesting similar usage efficiencies for both CD4 and CCR5. However, in the presence of MVC both of the incompletely inhibited Envs required much higher levels of CCR5 to achieve detectable levels of entry. This was particularly the case for 1684-E-7 Env that displayed the highest MPI to MVC, which supports the conclusion that the magnitude of the reduced MPI to MVC is determined by the efficiency of the interaction with MVC-bound CCR5.

### Amino acid determinants of HIV-1 strains with reduced MPIs to MVC map to the V3 gp120 region and are subject-specific in the C-HIV cohort

**Fig. 4:**
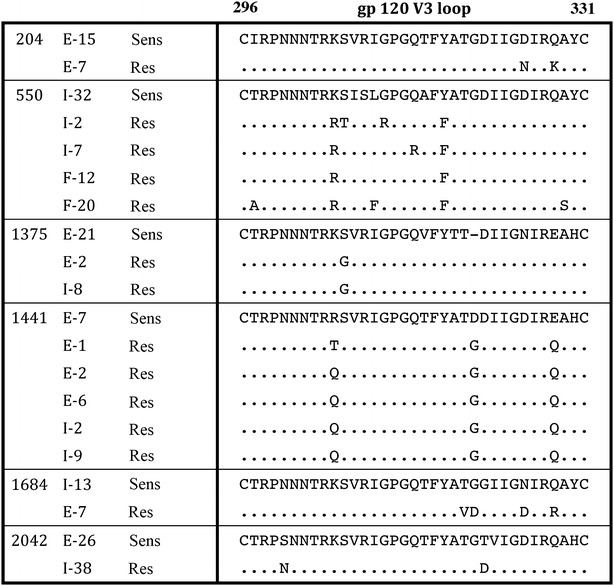
V3 loop mutations distinguish Env clones from the same subjects that display complete or incomplete inhibition of HIV-1 by MVC. The V3 loop amino acid sequences of Env clones displaying incomplete inhibition by MVC and one representative Env clone displaying complete inhibition are shown from 6 subjects. *Dots* indicate residues identical to the representative sensitive clone and *dashes* indicate gaps. The numbering is based on the HXB2 Env amino acid sequence. Sens: Representative Env clone displaying complete inhibition by MVC, Res: Envs clones that display incomplete inhibition by MVC

**Fig. 5:**
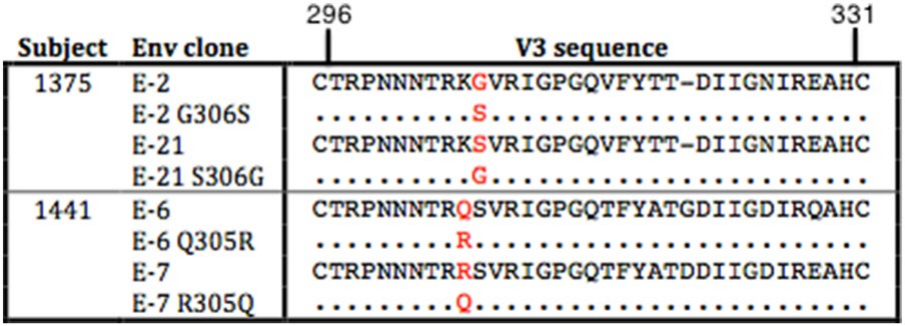
V3 loop amino acid sequence of Env mutants. The V3 loop sequences from the parental and mutant Env clones from two individuals are shown. The amino acid alterations are shown in *red*. *Dots* indicate residues identical to the parental strain and *dashes* indicate gaps. The numbering is based on the HXB2 Env amino acid sequence

As previous studies of B-HIV Envs have shown, changes in the gp120 V3 loop contribute to the emergence of resistance to CCR5-antagonist HIV-1 entry inhibitors [28, 29, 58]. To investigate the determinants that contribute to reduced MPIs to MVC in treatment-naïve C-HIV infected subjects, we next analyzed V3 sequences of Envs that displayed plateaus below 100% in 293-Affinofile CD4^med^/CCR5^hi^ cells, compared to their corresponding genetically-related Envs that were completely inhibited by MVC. For all subjects, the incompletely inhibited Envs displayed V3 sequences that differed from completely inhibited Envs recovered from the same subjects (Fig. 4). For the subsequent analyses we focussed on subjects 1375 and 1141, primarily because the incompletely inhibited Envs from these subjects displayed the lowest MPIs to MVC (Fig. 2) and had relatively consistent and/or discrete patterns of V3 changes whose functional properties could be more readily interrogated by mutagenesis studies (Fig. 4). Incompletely inhibited Envs from subject 1375 have a Gly residue at Env position 306, whereas completely inhibited Envs have Ser at this position. Similarly, completely inhibited Envs from subject 1441 possess Arg at position 305, whereas the majority of the incompletely inhibited Envs from subject 1441 have Gln at position 305. Although distinguishing amino acid changes also occurred at positions 320 and 328 for subject 1441, we focussed on the distinguishing change at position 305 because previous studies have shown that mutations at this position can contribute to CCR5 antagonist resistance by clinical HIV-1 subtype C strains [51, 53]. To determine if these V3 loop mutations conferred incomplete MVC inhibition, we created residue swap mutants introducing Ser at position 306 for the incompletely inhibited Env 1375-E-2; Gly at position 306 for the completely inhibited 1375-E-21; Gln at position 305 for the completely inhibited Env 1441-E-7; and Arg at position 305 for the incompletely inhibited Env 1441-E-6 (Fig. 5). The mutant Envs retained an R5 phenotype when tested for infectivity on NP2-CD4/CCR5 and NP2-CD4/CXCR4 cells (data not shown).

**Fig. 6:**
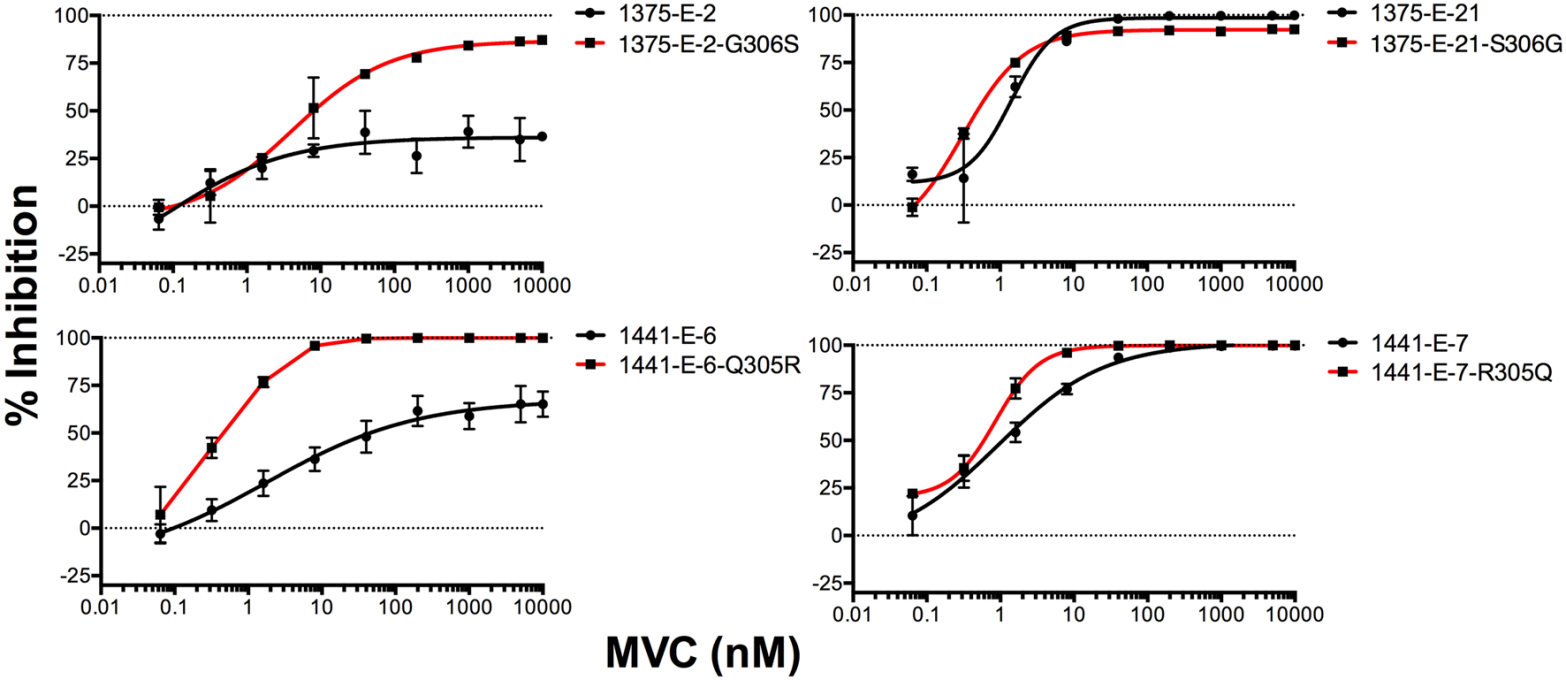
V3 loop mutations are necessary but not sufficient for incomplete MVC inhibition phenotypes of HIV-1 subtype C Envs. Luciferase reporter viruses pseudotyped with parental and mutant Envs from subjects 1375 and 1441 were used to infect 293-Affinofile CD4^med^/CCR5^hi^ cells in the presence of increasing concentrations of MVC. Virus inhibition curves were constructed as described in the “Methods”. The *data points* represent the mean and standard error of triplicate wells from one representative experiment of three independent experiments

We next generated luciferase reporter viruses pseudotyped with the Env mutants, and performed MVC sensitivity assays in 293-Affinofile CD4^med^/CCR5^hi^ cells (Fig. 6). Introduction of Gly306 into the completely inhibited 1375-E-21 Env led to a modest reduction in MPI from 98.3 ± 1.9 (mean % ± standard deviation) to 92.2 ± 0.5; whilst introduction of Ser306 into the incompletely inhibited 1375-E-2 Env led to an increase in MPI from 36.1 ± 2.9 to 86.7 ± 2.4 and thus, partial restoration of MVC sensitivity. Similarly, introduction of Gln305 into the completely inhibited 1441-E-7 Env made no appreciable difference in MPI (101.3 ± 1.6 to 99.9 ± 0.6), and introduction of Arg305 into the incompletely inhibited 1441-E-6 Env completely restored MVC sensitivity (67.4 ± 4.0 to 100.4 ± 1.2). These results suggest that for the Envs tested, V3 loop mutations are necessary but not sufficient for baseline incomplete HIV-1 inhibition by MVC, and that these mutations are strain specific.

Multiple studies have shown that the primary determinants of HIV-1 resistance to CCR5 antagonists lie within the V3 loop of gp120 [28–30, 59–63]. In agreement with these studies, our results show that single V3 loop mutations are necessary for incomplete inhibition by MVC for the C-HIV Envs studied. However, the single V3 loop mutations were not sufficient to confer the incomplete inhibition phenotype suggesting that additional mutations outside of V3 may be required. This has been observed in other CCR5 antagonist resistant strains [29, 57, 58]. Furthermore, we showed that amino acid mutations important for incomplete MVC inhibition were strain specific, which is commonly observed amongst CCR5 antagonist resistant strains generated in vitro and in vivo [28, 29, 57, 58, 64].

## Conclusions

Our results demonstrate the presence of HIV-1 Env glycoproteins that display incomplete inhibition to the CCR5 antagonist MVC in a proportion of the ART-naïve subtype C infected individuals from our cohort. The incomplete inhibition profiles we describe are similar in mechanism to those seen in Envs isolated from individuals who fail MVC-containing therapy, which most likely involves the use of the MVC-modified form of CCR5 for entry. Furthermore, similar to genuine MVC-resistant strains, we were able to map residues critical for incomplete inhibition to the V3 loop of gp120. In agreement with our previous studies [29, 47] and those of other investigators [33, 34] on CCR5 antagonist resistance, we demonstrated that incomplete MVC inhibition profiles are more readily apparent in 293-Affinofile cells induced to express high levels of CCR5. Whether the C-HIV strains described here with incomplete MVC inhibition may act as scaffolds for the generation of genuine MVC resistance during MVC containing ART regimens is a possibility that remains to be determined by further studies.

## Additional files

**Additional file 1: Table S1.** C-HIV Env clones used and their GenBank accession numbers.

**Additional file 2: Table S2.** Alternative coreceptor usage of Envs displaying incomplete HIV-1 inhibition by MVC compared to a representative Env from each subject that displays complete inhibition.
