# Supplementary material for: Frequency and Env determinants of HIV-1 subtype C strains from antiretroviral therapy-naive subjects that display incomplete inhibition by maraviroc
Source: Retrovirology. 2016 Nov 3;13:74. doi: 10.1186/s12977-016-0309-2 (PMC5093974; doi:10.1186/s12977-016-0309-2)
Supplement: Supplementary file 1 — Additional file 1: Table S1. C-HIV Env clones used and their GenBank accession numbers. [file 12977_2016_309_MOESM1_ESM.pdf]

Supplementary Table 1. C-HIV Env clones used and their GenBank accession numbers

Subject	Time point (E=enrolment; I=intermediate; F=final)	Clone number	GenBank Accession Number
**204** n=13	E	7	HQ708023
		10	HQ708019
		12	HQ708020
		15	HQ708021
		17	HQ708022
	I	6	HQ708032
		9	HQ708033
		10	HQ708031
	F	3	HQ708025
		4	HQ708026
		5	HQ708027
		8	HQ708029
		10	HQ708024
**258** n=12	E	5	HQ708050
		6	HQ708051
		20	HQ708047
		23	HQ708048
	I	1	HQ708058
		2	HQ708059
		6	HQ708061
		9	HQ708062
	F	1	HQ708052
		2	HQ708053
		20	HQ708054
		24	HQ708056
**455** n=16	E	23	HQ708063
		24	HQ708064
		27	HQ708065
		28	HQ708066
	I	2	HQ708073
		3	HQ708076
		5	HQ708077
		9	HQ708078
		22	HQ708074
		24	HQ708075
	F	1	HQ708067
		2	HQ708068
		5	HQ708071
		9	HQ708072
		22	HQ708069
		23	HQ708070

Subject	Time point (E=enrolment; I=intermediate; F=final)	Clone number	GenBank Accession Number
**513** n=11	E	4	HQ708080
		8	HQ708081
	I	1	HQ708089
		5	HQ708093
		8	HQ708094
		16	HQ708090
	F	1	HQ708082
		4	HQ708087
		22	HQ708084
		26	HQ708085
		27	HQ708086
**550** n=13	E	3	HQ708097
		8	HQ708098
		13	HQ708095
		20	HQ708096
	I	2	HQ708104
		7	HQ708108
		30	HQ708105
		31	HQ708106
		32	HQ708107
	F	1	HQ708099
		9	HQ708103
		19	HQ708100
		20	HQ708101
**574** n=15	E	6	HQ708113
		11	HQ708109
	I	4	HQ708126
		12	HQ708122
		13	HQ708123
		14	HQ708124
		15	HQ708127
	F	1	HQ708114
		4	HQ708118
		5	HQ708119
		6	HQ708120
		8	HQ708121
		10	HQ708115
		12	HQ708116
		15	HQ708117
**805** n=7	E	15	HQ708127
		29	HQ708128
		31	HQ708129
	I	2	HQ708130
		7	HQ708132
		8	HQ708133
		34	HQ708131

Subject	Time point (E=enrolment; I=intermediate; F=final)	Clone number	GenBank Accession Number
**858** n=17	E	1	HQ708135
		2	HQ708136
		4	HQ708141
		8	HQ708142
		22	HQ708137
		23	HQ708138
		38	HQ708140
	I	1	HQ708149
		5	HQ708152
		8	HQ708154
		10	HQ708150
		20	HQ708151
	F	3	HQ708146
		6	HQ708147
		9	HQ708148
		21	HQ708134
		26	HQ708144
**1109** n=7	E	42	HQ707834
		43	HQ707835
		46	HQ707836
		49	HQ707837
	I	9	HQ707849
		10	HQ707844
		19	HQ707847
**1114** n=14	E	1	HQ707850
		8	HQ707854
		9	HQ707855
		10	HQ7078**51**
	I	1	HQ707861
		4	HQ707863
		5	HQ707864
		6	HQ707865
		8	HQ707866
		10	HQ707862
	F	17	HQ707856
		18	HQ707857
		19	HQ707858
		33	HQ707859
**1136** n=12	E	1	HQ707867
		4	HQ707870
		11	HQ707868
		12	HQ707869
	I	3	HQ707880
		10	HQ707877
		11	HQ707878
		12	HQ707879
	F	7	HQ707876
		11	HQ707871
		12	HQ707872
		13	HQ707873

Subject	Time point (E=enrolment; I=intermediate; F=final)	Clone number	GenBank Accession Number
**1375** n=11	E	2	HQ707882
		3	HQ707885
		5	HQ707886
		20	HQ707883
		21	HQ707884
	I	2	HQ707892
		3	HQ707893
		8	HQ707895
		9	HQ707896
	F	13	HQ707888
		20	HQ707889
**1408** n=11	E	30	HQ707897
		31	HQ707898
		33	HQ707899
		35	HQ707901
		40	HQ707902
	I	31	HQ707904
		33	HQ707905
		34	HQ707906
		36	HQ707908
		37	HQ707909
		38	HQ707910
**1441** n=12	E	1	HQ707911
		2	HQ707912
		6	HQ707915
		7	HQ707916
	I	2	HQ707921
		3	HQ707922
		8	HQ707923
		9	HQ707924
	F	20	HQ707917
		24	HQ707918
		28	HQ707919
		32	HQ707920
**1503** n=11	E	11	HQ707925
		13	HQ707926
		22	HQ707928
	I	7	HQ707943
		11	HQ707939
		13	HQ707940
		27	HQ707942
	F	7	HQ707935
		9	HQ707937
		40	HQ7079**31**
		43	HQ707933

Subject	Time point (E=enrolment; I=intermediate; F=final)	Clone number	GenBank Accession Number
**1554** n=13	E	2	HQ707945
		3	HQ707946
		8	HQ707950
		10	HQ707944
	I	6	HQ707959
		7	HQ707960
		8	HQ707961
	F	4	HQ707956
		8	HQ707957
		10	HQ707951
		25	HQ707952
		26	HQ707953
		29	HQ707954
**1684** n=10	E	7	HQ707965
		9	HQ707966
		19	HQ707963
	I	13	HQ707972
		26	HQ707973
		29	HQ707974
		30	HQ707975
	F	8	HQ707971
		17	HQ707968
		22	HQ707969
**1689** n=10	E	6	HQ707977
		8	HQ707978
		9	HQ707979
	I	24	HQ707985
		25	HQ707986
		26	HQ707987
		28	HQ707988
	F	2	HQ707980
		5	HQ707983
		26	HQ707982
**1854** n=14	E	5	HQ707991
		8	HQ707992
		10	HQ707990
	I	3	HQ708001
		4	HQ708002
		6	HQ708005
		7	HQ708006
		20	HQ708000
	F	6	HQ707998
		8	HQ707999
		14	HQ707993
		17	HQ707994
		18	HQ707995
		21	HQ707996

Subject	Time point (E=enrolment; I=intermediate; F=final)	Clone number	GenBank Accession Number
**2042** n=11	E	15	HQ708007
		18	HQ708008
		26	HQ708009
	I	13	HQ708014
		21	HQ708015
		27	HQ708016
		38	HQ708017
	F	24	HQ708010
		25	HQ708011
		26	HQ708012
		28	HQ708013
**2253** n=9	E	21	HQ708037
		22	HQ708038
	I	21	HQ708041
		23	HQ708043
		27	HQ708044
		28	HQ708045
		32	HQ708046
	F	29	HQ708035
		36	HQ708036
