# Supplementary material for: Frequency and Env determinants of HIV-1 subtype C strains from antiretroviral therapy-naive subjects that display incomplete inhibition by maraviroc
Source: Retrovirology. 2016 Nov 3;13:74. doi: 10.1186/s12977-016-0309-2 (PMC5093974; doi:10.1186/s12977-016-0309-2)
Supplement: Supplementary file 2 — Additional file 2: Table S2. Alternative coreceptor usage of Envs displaying incomplete HIV-1 inhibition by MVC compared to a representative Env from each subject that displays complete inhibition. [file 12977_2016_309_MOESM2_ESM.pdf]

Supplementary Table 2. Alternative coreceptor usage of Envs displaying incomplete HIV-1 inhibition by MVC compared to a representative Env from each subject that displays complete inhibition

Patient ID	Env clone		Coreceptor usage			
	T	#	CCR5	CCR3	FPRL1	CCR8
204	E-15	Sens	++	-	++	-
	E-7	Res	++	+	++	-
550	I-32	Sens	+++	-	+++	-
	I-2	Res	+++	+++	+++	-
	I-7	Res	+++	+++	+++	-
	F-12	Res	+++	+++	+++	-
	F-20	Res	+++	++	+++	-
1375	E-21	Sens	+++	-	+++	+
	E-2	Res	+++	+	+++	++
	I-8	Res	+++	+	+++	+
1441	E-7	Sens	+++	-	++	-
	E-1	Res	+++	-	++	+
	E-2	Res	+++	-	++	+
	E-6	Res	+++	+	+++	+
	I-2	Res	+++	+	++	++
	I-9	Res	+++	+	+++	++
1503	E-11	Sens	+++	-	-	-
	F-7	Res	+++	-	-	-
1684	I-13	Sens	++	+	++	-
	E-7	Res	+++	++	+++	+
1854	E-8	Sens	+++	++	+++	++
	I-7	Res	+++	++	+	-
2042	E-26	Sens	+++	-	++	-
	I-38	Res	++	+	++	-

The level of virus entry into NP2-CD4 cells expressing CCR3, FPRL1 or CCR8 was scored as - (<5 fold above background), + (5-50 fold above background), ++ (50-300 fold above background), or +++ (>300 fold above background). These data were sourced and are described in detail in a previous study (Cashin et al., Retrovirology, 2013, 10:98). The results for virus entry into NP2-CD4 cells expressing CCR5, using equivalent infectious units of virus inoculum, are shown alongside these data for comparison. E, I and F refer to Envs cloned from plasma obtained at study enrolment, approximately 1 year later (intermediate), and approximately 3 years after enrolment (final) , respectively. Sens; Envs that are completely inhibited by MVC. Res; Envs that display incomplete inhibition by MVC.
